# Estimating and interpreting migration of Amazonian forests using spatially implicit and semi‐explicit neutral models

**DOI:** 10.1002/ece3.2930

**Published:** 2017-05-05

**Authors:** Edwin Pos, Juan Ernesto Guevara Andino, Daniel Sabatier, Jean‐François Molino, Nigel Pitman, Hugo Mogollón, David Neill, Carlos Cerón, Gonzalo Rivas‐Torres, Anthony Di Fiore, Raquel Thomas, Milton Tirado, Kenneth R. Young, Ophelia Wang, Rodrigo Sierra, Roosevelt García‐Villacorta, Roderick Zagt, Walter Palacios Cuenca, Milton Aulestia, Hans ter Steege

**Affiliations:** ^1^ Ecology and Biodiversity GroupUtrecht UniversityUtrechtThe Netherlands; ^2^ Group of Dynamic BiodiversityNaturalis Biodiversity CenterLeidenThe Netherlands; ^3^ Department of Integrative BiologyUniversity of California, BerkeleyBerkeleyCAUSA; ^4^ IRD, UMR AMAPMontpellierFrance; ^5^ The Field MuseumChicagoILUSA; ^6^ Center for Tropical ConservationNicholas School of the EnvironmentDuke UniversityDurhamNCUSA; ^7^ Endangered Species CoalitionSilver SpringMDUSA; ^8^ Universidad Estatal AmazónicaPuyoEcuador; ^9^ Escuela de Biología Herbario Alfredo ParedesUniversidad Central Herbario Alfredo ParedesQuitoEcuador; ^10^ Colegio de Ciencias Biológicas y Ambientales and GalápagosAcademic Institute for the Arts and SciencesUniversidad San Francisco de QuitoQuitoEcuador; ^11^ Department of AnthropologyUniversity of Texas at AustinAustinTXUSA; ^12^ Iwokrama International Programme for Rainforest ConservationGeorgetownGuyana; ^13^ GeoISQuitoEcuador; ^14^ Geography and the EnvironmentUniversity of Texas at AustinAustinTXUSA; ^15^ Northern Arizona UniversityFlagstaffAZUSA; ^16^ Institute of Molecular Plant SciencesUniversity of EdinburghEdinburghUK; ^17^ Royal Botanic Garden of EdinburghEdinburghUK; ^18^ Tropenbos InternationalWageningenThe Netherlands; ^19^ Herbario Nacional del EuadorUniversidad Técnica del NorteQuitoEcuador; ^20^ Herbario Nacional del EcuadorQuitoEcuador; ^21^ Systems EcologyFree University AmsterdamThe Netherlands; ^22^ Department of Wildlife Ecology and Conservation110 Newins‐Ziegler HallUniversity of FloridaGainesvilleFL32611

**Keywords:** betadiversity, migration, neutral theory, parameter estimation, species composition, species diversity

## Abstract

With many sophisticated methods available for estimating migration, ecologists face the difficult decision of choosing for their specific line of work. Here we test and compare several methods, performing sanity and robustness tests, applying to large‐scale data and discussing the results and interpretation. Five methods were selected to compare for their ability to estimate migration from spatially implicit and semi‐explicit simulations based on three large‐scale field datasets from South America (Guyana, Suriname, French Guiana and Ecuador). Space was incorporated semi‐explicitly by a discrete probability mass function for local recruitment, migration from adjacent plots or from a metacommunity. Most methods were able to accurately estimate migration from spatially implicit simulations. For spatially semi‐explicit simulations, estimation was shown to be the additive effect of migration from adjacent plots and the metacommunity. It was only accurate when migration from the metacommunity outweighed that of adjacent plots, discrimination, however, proved to be impossible. We show that migration should be considered more an approximation of the resemblance between communities and the summed regional species pool. Application of migration estimates to simulate field datasets did show reasonably good fits and indicated consistent differences between sets in comparison with earlier studies. We conclude that estimates of migration using these methods are more an approximation of the homogenization among local communities over time rather than a direct measurement of migration and hence have a direct relationship with beta diversity. As betadiversity is the result of many (non)‐neutral processes, we have to admit that migration as estimated in a spatial explicit world encompasses not only direct migration but is an ecological aggregate of these processes. The parameter *m* of neutral models then appears more as an emerging property revealed by neutral theory instead of being an effective mechanistic parameter and spatially implicit models should be rejected as an approximation of forest dynamics.

## 1 Introduction

Whether stochastic or deterministic processes govern species distribution has been a long‐standing debate, starting with the equilibrium versus nonequilibrium theories more than 25 years ago (DeAngelis & Waterhouse, 1987). The Unified Neutral Theory of Biodiversity and Biogeography (UNTB—Hubbell, 2001) refueled this discussion (Adler, HilleRisLambers, & Levine, 2007; Alonso, Etienne, & McKane, 2006; Clark, 2009; Leigh, 2007; McGill, Maurer, & Weiser, 2006; Purves & Turnbull, 2010). Prior to this debate, the main accepted view of population dynamics was of a niche‐based origin, that is, species being specifically adapted to certain environments where they could thrive, while outcompeted elsewhere. Processes as competitive exclusion (Gause, 1934; Hardin, 1960) and niche partitioning (Grinnell, 1917, 1924; Patten & Auble, 1981) were believed to be the main drivers of differences in species composition. Actual niches occupied by species were thought to be determined by specific suits of adaptations for certain environments and biotic interactions among species (Hutchinson, 1959). This combination of interspecific differences and environmental heterogeneity allowed for coexistence. In contrast, the UNTB is neither based on such interspecific differences nor environmental heterogeneity. It assumes that all individuals are ecologically equivalent in terms of demographic events such as birth and death, but also in rates of migration and their probability of speciation. As a result, the main differences in species composition are simply based on stochastic processes, resulting from ecological equivalence. It was not a fully novel approach, however, as the model of Island Biogeography by MacArthur and Wilson (1967) was also truly neutral in its mathematical foundations treating species equivalent in demographics, even though the authors still regarded species as having distinct niches in real life. Much work on neutral theory had already been developed in population genetics, some implicit, such as the Island Model (Wright, 1943), and others explicit such as the Stepping Stone model (Kimura & Weiss, 1964). The UNTB relies heavily on these models of genetic differentiation between communities, with the neutral theory of molecular evolution (Kimura, 1983) obviously being one of its pillars (Hubbell, 2001). Many criticized the UNTB (Duivenvoorden, Svenning, & Wright, 2002; Magurran & Henderson, 2003; Pitman, Terborgh, & Silman, 2001, 2002; Terborgh & Foster, 1996; Tuomisto, Ruokolainen, & Yli‐Halla, 2003; Valladares, Wright, & Lasso, 2000) and many supported it (de Aguiar, Baranger, Baptestini, Kaufman, & Bar‐Yam, 2009; Bell, 2000; Chave, 2004; Condit et al., 2002; Volkov, Banavar, Hubbell, & Maritan, 2003). Today, many ecologists agree that both deterministic and neutral processes play a role in determining species composition (Barot, 2009; Gravel, Canham, Beaudet, & Messier, 2006; McGill, 2010; McGill & Nekola, 2010). To study their relative importance, models are often used to investigate whether communities behave neutrally or not. An important question still remaining is how to parameterize neutral models. Suggestions for estimating two of the core parameters of Hubbell's neutral model, speciation and migration, have been proposed over the years, and the importance of parameter estimation has been discussed previously (Beeravolu, Couteron, Pélissier, & Munoz, 2009). These studies concentrated, however, specifically on the difference between estimating from a single (large) sample or multiple samples in a spatially continuous landscape. They did not focus on the role of spatial relationships, that is, the effect of distance between plots when estimating migration. We feel this effect of distance is important because space and migration can be incorporated in two different ways, either spatially implicit (Caswell, 1976; Hubbell, 2001) or spatially explicit (e.g., Chave & Leigh, 2009; Condit et al., 2002; Horvát, Derzsi, Néda, & Balog, 2010; O'Dwyer & Green, 2010). Models of the first kind work on the assumption of a panmictic system. They disregard the spatial position of individuals within each community as there is only one migration parameter *m*, determining whether a recruit is from the regional or local species pool, but there is no within‐community dispersal limitation. Even though such models show good fits, the existence of such a panmictic community is unlikely, due to the physical dispersal ability of individuals versus the size of many communities (Kimura & Weiss, 1964). In contrast, spatially explicit models consider the metacommunity rather as the sum of a number of local communities, between which there exists an explicit spatial relationship. The first models, where the spatial position of each individual was explicitly modeled, were based on a discrete grid‐like structure, each cell containing an individual which could disperse either to neighboring cells (Durrett & Levin, 1996; Zillio, Volkov, Banavar, Hubbell, & Maritan, 2005) or to other regions by implementing different dispersal kernels (Chave & Leigh, 2002; Condit et al., 2002). However, while there are quite some analytical solutions for the implicit models, only few exist for the explicit versions such as developed by O'Dwyer and Green (2010) by applying principles from physics.

Comparisons show that, although spatially explicit models should approximate the real world better, spatially implicit models provide better fits to empirical data (Etienne & Rosindell, 2011; Rosindell, Hubbell, & Etienne, 2011). Hence, the latter are more often used when estimating migration, even though field data comes from a spatially explicit reality. In this study, we therefore extend the comparison of estimation methods toward the practical ability of these methods to estimate migration from simulated datasets based on both spatially implicit and spatially semi‐explicit models. We focus on five different parameter estimation methods: (1) a sampling formula by Etienne (2005), (2) the Inference method by Jabot, Etienne, and Chave (2008), (3) the *G*_st_ statistic adopted from population genetics by Munoz, Couteron, and Ramesh (2008), (4) the two‐stage sampling formula by Etienne (2009b), which is an extension on the two‐stage estimation method by Munoz, Couteron, Ramesh, and Etienne (2007), and (5) a method by Chisholm and Lichstein (2009) based on plot geometry and absolute dispersal distances. A summary of the different estimation methods can be found in the Appendix S1. For the interested and more mathematically oriented reader, we refer to the original papers, as here we are focusing on the use of the methods rather than their exact mathematical derivation. Our first goal was to perform a sanity check on each method. They should at least be able to recover parameter estimates from models on which they are based. Our second and main goal was to establish whether these methods are also robust, that is, whether they are able to accurately recover parameters when performed on models a bit different from the models on which they are based. For this, we apply them to a spatially semi‐explicit model in which migration can either be from a hypothetical metacommunity or from adjacent plots. Our last and third goal was to apply each method to empirical field data. For this, we use three different independent field datasets: Guyana/Suriname, French Guiana and Ecuador, which are highly distinct in their forest dynamics (Malhi et al., 2006). Using field data and data from spatially implicit and semi‐explicit simulations, we hope to reach a broad public of ecologists working on similar problems.

## 2 Methodology

### 2.1 Comparison of model parameter estimation

Each parameter estimation method, as described above, was used to generate an estimation of migration for a number of situations using spatially implicit, (semi‐)explicit simulated, and field datasets. Results were compared from the simulated datasets in terms of their ability to accurately describe migration as parameterized to construct the datasets. After using the simulated datasets, we turned to the actual field data, having multiple local communities assumed to be a sample from the larger metacommunity for which migration was also estimated using the same estimation methods. Etienne's (2005) sampling formula and the Inference method of Jabot et al. (2008) were both tested using the TeTame freeware version 2.1 http://chave.ups-tlse.fr/projects/tetame.htm. Etienne's (2009b) two‐stage sampling method was tested using the PARI/GP environment (“PARI/GP version 2.4.3”, 2008). Chisholm & Lichsteins's method was tested using MATLAB (2004), and the *G*_st_ statistic was computed using the package *untb* (Hankin, 2007) in the R environment (R Core Team, 2014). Other R packages used were *Quantreg, Vegan*,* Labdsv*, and *FasianOptions* (Koenker, 2013; Oksanen et al., 2013; Roberts, 2013; Wuertz et al., 2013). All R scripts used are available upon request from main author.

### 2.2 Metacommunity simulation

For both spatially implicit and explicit simulations, the first step was to create the larger metacommunity. The relative abundance distribution of tree species in the Amazonian forests shows a nearly exact fit with Fisher's logseries (Hubbell et al., 2008; ter Steege et al., 2013). We therefore used this relationship and the related number of species for a given abundance (Fisher, Corbet, & Williams, 1943) to derive the relative abundance distribution from the expected number of species (*S*) and individuals (*N*) in the metacommunity, given by Φ_*n*_ = α*x* ^*n*^/*n*. Here, Φ_*n*_ is the number of species with *n* individuals; α is Fisher's α and *x* is given by *N*/(*N* + α) (*N* being the number of individuals in the total sample and *x* being asymptotically equal to 1 with large sample sizes). We created three different metacommunities: two for the simulated spatially implicit datasets and one for the spatially semi‐explicit dataset. Because of the observed difference between the Guianas and Ecuador in terms of diversity and composition (ter Steege et al., 2013, fig S10) and the regions being separated by a large geographical distance, we created two different metacommunities for the spatially implicit simulations related to these two regions rather than one large metacommunity. They are hereafter referred to as MC‐high and MC‐low, respectively (metacommunity high and low diversity). ter Steege et al. (2013) estimated mean tree densities for all species per degree grid cell and by fitting the mean rank abundance curve to Fisher's logseries distribution estimated the total amount of species to be expected by country (ter Steege et al., 2013 fig S10). We adopted these figures to construct MC‐low (20, 191, 600, 511 individuals and 4,582 species) and MC‐high (5, 611, 001, 426 and 6,834), for details on both see the Supporting information. For the simulated spatially explicit dataset, a separate metacommunity was constructed using the same methods based on the Reserva Ducke forest, with 5.5 million trees and a Fisher's α of 272 (ter Steege et al., in press), hereafter referred to as MC_spatial. The logseries for each community was constructed starting from the left tail (the most dominant species). The fixed parameters *alpha* and *x* were first calculated from the number of individuals (*N*) and species (*S*), after which the maximum dominance according to Fisher's logseries for all species is calculated, which is then given the first rank. For each subsequent rank, the predicted number of species is then calculated until all species are given a rank and all individuals are distributed.

### 2.3 Spatially implicitly simulated data

For the spatially implicit datasets, we used the exact same sampling procedure as proposed by Hubbell in the original UNTB. Each time step, one individual dies, which is replaced by an individual having an ancestor either in the local community (with probability 1 − *m*) or from the metacommunity (with probability *m*). The identity of the recruit is then only dependent on its relative abundance in the respective community. Datasets based on GS and FG (67 and 63 plots) were sampled from the MC‐low assuming they share the same metacommunity and the dataset based on EC (72 plots) from MC‐high. Sampling of the local communities was repeated for a range of migration parameters (see Table S1). For details on the number of time steps used see the Appendix S2. After the construction of the simulated datasets, migration was estimated using the above‐mentioned estimation methods.

### 2.4 Spatially semi‐explicitly simulated data

Spatially semi‐explicit simulations were carried out by modeling a lattice of 20 × 20 plots, each with 500 individuals. We assume no spatial explicit arrangement of individuals within a plot. Taking a random sample from the metacommunity creates the forest at time *t*_0._ Each time step (*t*_+1_) one individual from each plot to be replaced was chosen at random from the MC_spatial metacommunity, and this was repeated for 10,000 time steps. Recruitment was generated from either of three sources: (1) migration from adjacent plots (*m.adj*), (2) migration from the MC_spatial metacommunity (*m.meta*), or (3) local recruitment (1−(*m.adj + m.meta)* = *1 *− *m*). According to studies on long‐distance dispersal of seeds (LDD), the majority of seeds (>99%) often fall within ca. 100 m of their origin (Nathan & Muller‐Landau, 2000), depending on among others, seasonal conditions, wind speed, turbulence initiated by the canopy, and particle fall velocity, which is obviously also affected by seed mass and shape (Bohrer, Katul, Nathan, Walko, & Avissar, 2008; Maurer, Bohrer, Medvigy, & Wright, 2013). As the plots from the field data used in this study are 1 ha in size, it is reasonable to assume that migration either does not occur but there is local recruitment, or there is migration mostly from adjacent plots when the tree of origin would be on the edge of a plot, with occasionally seeds ending up further away. Hence, this subdivision in dispersal categories using a discrete probability mass function seems a likely approximation of the actual dispersal of individuals and allows for much faster calculations by the computer. Values for both *m.adj* and *m.meta* were based on an arbitrary division of the range of migration used for the spatially implicit simulations (see also Table 2).

### 2.5 Species composition of field data

Three different sets of field data from the Amazon Tree Diversity Network (ter Steege et al., 2013) were used for analysis. Two sets belong to the Guiana Shield: Guyana/Suriname combined and French Guiana, and the third set contains data from forests in Ecuador. Hereafter, they are referred to as GS, FG, and EC, respectively. All three sets are completely independent and nonoverlapping (Pos et al., 2014). Datasets are composed of 63–72 one‐hectare plots with all trees ≥10 cm DBH inventoried. Species names of all datasets were standardized with the W3 Tropicos database within each dataset, using TNRS (Boyle et al., 2013), as described in more detail in ter Steege et al. (2013). The EC dataset has 72 plots of 1 ha, yielding 34,544 individuals and 2,021 morphospecies. The GS and FG datasets are composed of 67 and 63 one‐hectare plots, respectively. In GS, 37,446 individual trees were distributed among 1,042 morphospecies, and FG had 35,075 individuals belonging to 1,204 morphospecies.

## 3 Results

### 3.1 Comparing parameter estimation methods: spatially implicit and explicit

**Figure 1:**
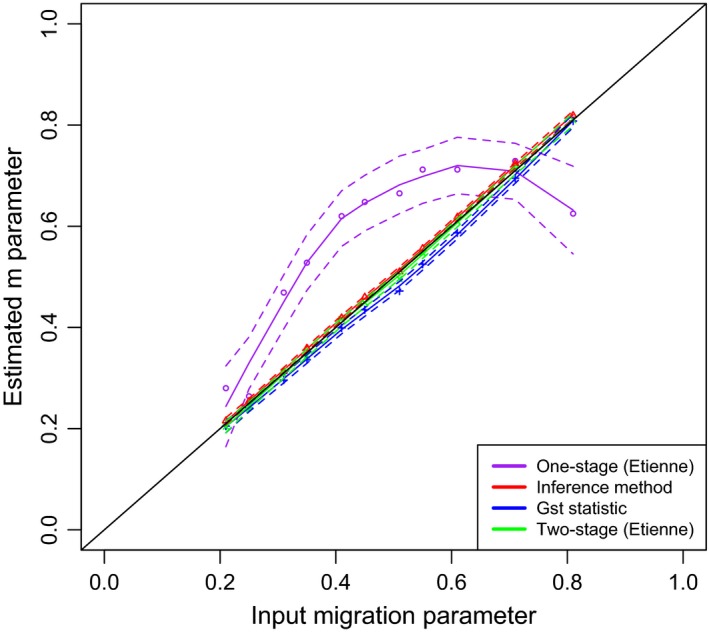
LOESS regressions of the migration parameter used for input versus the estimated migration from the spatially implicit simulations. Results from each method indicated by color with broken lines indicating the 95.5% confident interval, polynomial degree and span used for the LOESS regression was 2 and .75, respectively

**Figure 2:**
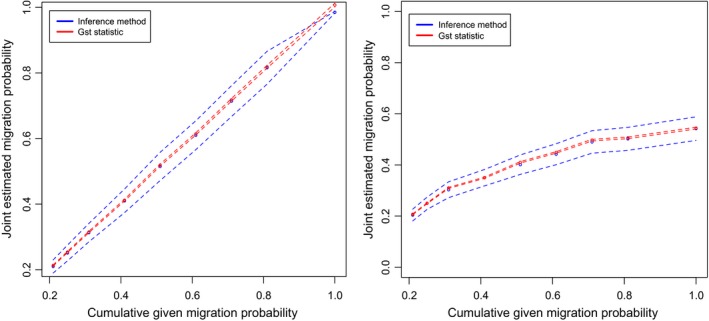
Given joint migration probability with either migration predominantly coming from the metacommunity (left) or from adjacent plots (right) plotted against the estimated joint migration by both the Inference method (blue) and *G*_st_ statistic (red). Broken lines indicate the estimation plus or minus the standard deviation of the average over all plots used in the simulation. It is clear that when migration mostly comes from the metacommunity, both estimation methods are very accurate, and when migration from adjacent plots is dominant, both estimation methods are underestimations

Sanity checks on each method showed that the Inference method and *G*_st_ statistic were able to approximate the complete range of migration parameters based on each different field dataset accurately. Etienne's one‐stage sampling method showed larger deviations. The two‐stage sampling by Etienne was only used for the spatially implicit dataset based on EC due to extreme long computation time (see details in the Appendix S1) but also generated accurate estimations. Average difference between given and estimated migration was .08, .007, .02, and .004 for the *G*_st_ statistic, Inference method, Etienne's one‐stage sampling, and Etienne's two‐stage sampling, respectively (see Table 1 for a summary and Table S1 for details). All methods except the one‐stage sampling by Etienne thus showed very good accuracy when given migration parameters were plotted against the estimated migration (Figure 1). Next, we tested the robustness of each of the methods when applied to slightly different models. Etienne's one‐stage sampling formula was not used for estimating data from spatially explicit simulation because of the larger deviations found with the spatially implicit simulations. The corrected plot geometry method was also excluded because estimation of migration would be constant over the range of parameters used. The two‐stage estimation method by Etienne was also not used due to practical limitations as explained earlier. Hence, we were only able to use the Inference method and *G*_st_ statistic. The migration estimates from the spatially semi‐explicit simulations were the additive effect between migration from the adjacent plots and the metacommunity (Table 2). As both methods generate a single migration value, they were only able to estimate the *joint migration* probability. As example, in one of the simulated sets, the parameters were set such that 1% of replacements were drawn from the eight cells surrounding the cell in which an individual died and 20% of replacements are drawn from the metacommunity surrounding these adjacent cells (*m.adj* of .01 and *m.meta* of .20, dataset 3). Both the *G*_st_ statistic and the Inference method estimated a migration probability of .21, indicating that these probabilities are additive in the estimation, and it is still unknown whether migration is from close by or far away. Estimation of the joint migration probability was only accurate when migration from the metacommunity was higher than from the adjacent plots. In the contrasting situation (*m.adj* > *m.meta*), estimations were generally an underestimation of the joint migration probability (Table 2 and Figure 2).

**Table 1:** Summary of Table S1, with the mean difference between given and estimated migration (Δ*m*), using spatially implicit simulations. Results from the corrected plot geometry method by Chisholm & Lichstein are not shown as they yield a single value with a confidence interval shown in Table S1

Summary difference m.given versus m.est and range *SD* of estimations								
Dataset	One‐stage est.		Inference method		*G*_st_ statistic		two‐stage (Etienne)	
	Δ*m*	*SD* range	Δ*m*	*SD* range	Δ*m*	*SD* range	Δ*m*	*SD* range
Guyana/Suriname	.044	.032–.06	.0075	.009–.016	.0200	.043–.382	—	—
French Guiana	.071	.033–.060	.0078	.009–.016	.0240	.044–.325	—	—
Ecuador	.132	.022–.061	.0070	.008–.018	.0160	.043–.418	.004	.017–.046

**Table 2:** Estimates of migration based on a semi‐spatially explicit neutral model. Probability of migration was determined from adjacent plots (*m.adj*), the metacommunity (i.e., all other plots except the local and adjacent plots; *m.meta*) or the local plot. Number of plots was 400 with a runtime of 1e^8^ for all datasets

Spatial semi‐explicit									
Simulation parameters and yielded variables						Estimated migration			
dataset	Nr. sp.	Nr. sing	m.local	m.adj	m.meta	Inference method		*G*_st_ statistic	
						m2	*SD*	m3	*SD*
1	1,777	244	0	0	1.00	.990	.028	1.011	.0057
2	1,088	37	.79	.20	.01	.140	.015	0.156	.0012
3	1,529	142	.79	.01	.20	.209	.021	0.210	.0015
4	1,542	147	.75	.05	.20	.244	.024	0.247	.0017
5	1,282	73	.75	.20	.05	.200	.019	0.205	.0014
6	1,093	48	.69	.30	.01	.197	.019	0.215	.0013
7	1,609	169	.69	.01	.30	.310	.027	0.312	.0020
8	1,277	74	.65	.30	.05	.260	.023	0.270	.0017
9	1,077	50	.59	.40	.01	.254	.021	0.277	.0016
10	1,666	182	.59	.01	.40	.416	.034	0.419	.0024
11	1,315	97	.55	.40	.05	.325	.027	0.341	.0019
12	1,056	36	.49	.50	.01	.310	.028	0.330	.0019
13	1,690	186	.49	.01	.50	.512	.040	0.517	.0028
14	1,301	96	.45	.50	.05	.380	.032	0.400	.0023
15	1,706	187	.39	.01	.60	.615	.046	0.621	.0034
16	1,727	220	.29	.01	.70	.716	.050	0.721	.0039
17	1,748	224	.19	.01	.80	.819	.050	0.822	.0042

### 3.2 Parameter estimation from field data

**Figure 3:**
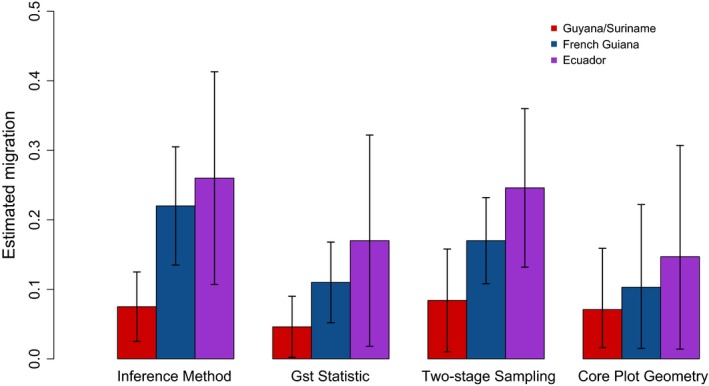
Estimated migration probability from each empirical dataset (GS, FG, and EC) for the Inference method, *G*_st_ statistic, Two‐stage Sampling estimation and the Corrected Plot Geometry method. For the first three methods, whiskers indicate standard deviation of the estimation. For the corrected plot geometry method, they are representative of the confidence interval for the estimation

The *G*_st_ statistic, Inference, and Etienne's two‐stage sampling formula were used to estimate migration from the three field datasets. Calculation of migration using the corrected plot geometry method was based on the following parameters: edge length of plot (*w*) 100 m for all three sets (as each plot is 1 ha) and mean absolute dispersal distances in the ranges 15–25 m for GS, 25–35 m for FG as it has more pioneer species in comparison with the first, and 40–50 m for EC as it is relatively comparable to the BCI plot in Panama having rich soils sustaining rapid dynamics (i.e., fast growth). This yielded migration parameters of .237 with a confidence interval (CI) of .182–.293, .344 (CI .293–.396), and .489 (CI .444–.533) for GS, FG, and EC, respectively (see also Table 3). After applying the correction as explained in the appendix, this was .071 (CI .055–.088), .103 (CI .088–.119), and .147 (CI .133–.160). Here, CI is given instead of *SD* as the corrected plot geometry method gives a single estimate depending on plot geometry and mean dispersal distance, and the CI is then related to the lower and upper limit of the dispersal range. In the same order (GS, FG, and EC), the *G*_st_ statistic yielded estimates of .046, .11, and .17 (*SD*: .044, .058, and .152). Using the Inference method, this was .075, .22, and .26 (*SD* .050, .085, and .153), and for Etienne's two‐stage sampling, this was .084, .170, and .246 (*SD* .074, .062, and .114), see also Table 3 and Figure 3.

**Table 3:** Parameter estimation for the three field datasets. For the corrected plot geometry method by Chisholm and Lichstein (2009), the following parameters were used: Guyana/Suriname *w* = 100, *d* = 15–25 m, French Guiana, *w* = 100 m, d = 25–35 m, Ecuador, *w* = 100 m and *d* = 40–50 m

Dataset	Inference method		*G*_st_ statistic		two‐stage (Etienne)		Cor. Plot Geometry	
	m2	*SD*	m3	*SD*	m4	*SD*	m5	CI
Guyana/Suriname	.075	.050	0.046	.044	.084	.074	.071	.055–.088
French Guiana	.22	.085	0.11	.058	.170	.062	.103	.088–.119
Ecuador	.26	.153	0.17	.152	.246	.114	.147	.133–.160

### 3.3 Comparing parameter estimation from field and simulated datasets

We implemented all migration parameters from the spatially implicit simulations in the spatially implicit model and compared the results of the relative abundance distribution (RAD), number of species and singletons (i.e., species with one individual), and Fisher's alpha generated from the simulations to the actual field data (Figures S5, S6 and Table 4). As example, for GS (having 1,042 morphospecies and 210 singletons), the simulated dataset based on the MC_low metacommunity using a migration of 0.046 (*G*_st_ statistic estimation from field data) yielded a total number of 826 species belonging to 41,875 individuals (67 plots times 625 individuals) with 83 singletons and an average Fisher's alpha of 146. When using .075 as the probability of migration (as estimated by the Inference method), this was 885 species, 69 singletons, and a Fisher's alpha of 158. For a migration of .084 (Etienne's two‐stage sampling method), this was 896 species, 78 singletons, and a Fisher's alpha of 164, and finally with a migration parameter of .071 (corrected plot geometry method), this was 801 species, 97 singletons, and 151 as a Fisher's alpha. Using the spatially implicit simulations with the estimation migration probabilities hence tended to show less species and a smaller amount of singletons than the actual field data, which was the case for FG and EC as well (see Table 4). For the comparison of RAD's from field data and simulations see Figures S5 and S6.

**Table 4:** Results from the spatially implicit model‐based estimates of *m* using the three separate field datasets

Dataset	Method	Migration	Metacommunity	Plots	Species	Singletons	Fisher's alpha
Guyana/Suriname	—	—	—	67	1,042	210	198
Guyana/Suriname	Inference method	.075	MC‐low	67	885	69	158
Guyana/Suriname	*G*_st_ statistic	.046	MC‐low	67	826	83	146
Guyana/Suriname	Two‐stage Etienne	.084	MC‐low	67	896	78	164
Guyana/Suriname	Cor. Plot Geometry	.071	MC‐low	67	801	97	151
French Guiana	—	—	—	63	1,204	208	177
French Guiana	Inference method	.220	MC‐low	63	1,045	113	197
French Guiana	*G*_st_ statistic	.110	MC‐low	63	964	105	179
French Guiana	Two‐stage Etienne	.170	MC‐low	63	975	116	188
French Guiana	Cor. Plot Geometry	.103	MC‐low	63	910	95	169
Ecuador	—	—	—	72	2,021	518	468
Ecuador	Inference method	.260	MC‐high	72	1,667	243	126
Ecuador	*G*_st_ statistic	.170	MC‐high	72	1,333	167	289
Ecuador	Two‐stage Etienne	.246	MC‐high	72	1,489	196	324
Ecuador	Cor. Plot Geometry	.147	MC‐high	72	1,373	196	292

Fisher's alpha was averaged over all plots; first row of each set shows actual field data.

## 4 Discussion

Most methods used for estimating migration rates of neutral models are based on Hubbell's original spatially implicit model or its derivations (Beeravolu et al., 2009; Etienne, 2005, 2009a,b; Jabot & Chave, 2009; Jabot et al., 2008; Munoz et al., 2007, 2008). This implicit approach contrasts strongly with reality for tropical trees, as the morphology of for example fruits and seeds, and also, different strategies play an important role in defining the average dispersal distance of plants (Gitay, Noble, & Connell, 1999; Swaine & Whitmore, 1988; Westoby, 1998). In addition, in real life, dispersal limitation is also not neutrally distributed among species. Although this disagreement is quite apparent, the inference of migration using such estimation methods is often done to study forest dynamics and the relative importance of niche versus neutral processes shaping communities. Here, we show that although the estimation methods we compared were able to correctly estimate migration from models of which they were derived, they fail to do so for models in which there is a spatially explicit relationship. For the spatially implicit simulations, the Inference method (Jabot et al., 2008) and *G*_st_ statistic (Munoz et al., 2007) yielded comparable results and were able to estimate migration very precisely (Table 1 and S1). The two‐stage sampling method by Etienne was only used for the spatially implicit datasets based on EC due to long computation time, but showed comparable results. The only exception was the one‐stage estimation method by Etienne (2005), which in particular for higher probabilities of migration showed a larger deviation (see also Figure 1). This method is based on the likelihood calculation of *P*[*D*|θ, *m*,* J*], the multivariate probability of observing a current specific species abundance distribution given the constraints of the parameters (see also Appendix S1). This in essence is the sum of all possible species–ancestry–abundance distributions. The problem that could occur here, although we did not test this explicitly, is that this may be a result of the way *m* is related to *I* by *m* = *I*/(*I* + *J* − 1) with *J* the size of the community. Hence, *I* = *m*(*j* − 1)/(1 − *m*), and when *m* approaches unity, I reaches infinity. Thus, as migration approaches one and I becomes increasingly large, the expression (4) from Etienne (2005) is reduced to become only dependent on one term, namely A = J. Intuitively, this means that all individuals in the community are a potential ancestor, thus coming from the metacommunity, and likelihood estimates of migration could potentially deviate substantially from what is given. Other problems might be caused by the way this method is implemented in the software as used in this study (R. S. Etienne, personal communication). Perhaps further study into this phenomenon could shed more light on these results.

When we turn to the semispatial explicit simulations, we see a different result. Each method yields only a single estimation for migration per sample. As such, it was obvious they would only estimate a joint migration probability instead of those from separate sources of migration. This total migration rate, however, could still be the “correct” total migration, if it would in fact measure actual migration or at least approximate it. Given that there is no spatial relationship in the model from which the methods are derived, however, we expected that estimation methods based on a spatial implicit reality would struggle to infer migration when this is larger from nearest neighbor communities than that from the larger metacommunity. Although intuitively this makes sense, as far as we know this has not been tested with actual large‐scale field data before nor has it been shown to what extent it would deviate using a quantitative modeling approach. Our results supported our expectation and showed that this joint estimation was accurate only when migration from the metacommunity was higher than from the adjacent plots. In contrast, if *m.adj > m.meta* which would be the normal situation in reality for tropical trees, estimations were consistently found to be an underestimation of the joint migration probability (see Table 2 and Figure 2). Although only the Inference method and *G*_st_ statistic were used for the latter, we assume given the earlier results on the spatially implicit simulations that the two stage sampling by Etienne would generate similar results.

Here, we show the consequences of using estimation methods based on a spatially implicit model to estimate migration from a spatially explicit reality. When the majority of migration is coming from the metacommunity, even spatially semi‐explicit simulations approach a spatially implicit reality. One could ask whether we would ever expect estimations of migration to be accurate when we are using spatially implicit models. Given the model's assumptions and rules, we think this would only be the case if the actual system approaches a spatially implicit system, that is, when there is no true spatial relationship between composition and geographical distance. In this case, these methods would estimate migration correctly (i.e., *m *= *m.adj *+ *m.meta*). In bryophytes, this may be the case, or at least the data were consistent with the predictions of the spatially implicit neutral model (Mota de Oliveira, ter Steege, Cornelissen, & Robbert Gradstein, 2009). When spores get in the upper wind layers, they are capable of traveling almost across the entire Amazon, although the majority of replacement will still be local recruits. In such a spatially implicit reality, each local community is considered a sample from the metacommunity, and how much it actually resembles the metacommunity depends on the migration parameter (estimated to .2 for the bryophytes). In Hubbell's original UNTB, species abundances deviate from the expected abundance (its proportional abundance in the metacommunity) because migration determines the time that ecological drift operates within the local community. In other words, the migration parameter determines differences in species diversity between the plot under consideration and the diversity of the total sample used for analysis and hence has a direct relationship with betadiversity found in the total sample. This is meaningful when estimating from neutral spatially implicit simulations, where the only relationship is that of migration between each plot and the metacommunity. When it comes to the real world, it is a different matter as betadiversity can be the result of many neutral and non‐neutral processes (Figure S9). As such, it also becomes apparent why the neutral model shows such good fits when estimating migration and implementing it in a neutral model, even though we know the world is not neutral. Migration as estimated from a spatially implicit model encompasses not only dispersal but is in fact an ecological aggregate of all processes determining betadiversity: dispersal, time, competition, habitat selectivity, predation, frequency‐dependent mortality, etc. It is the link between the (summed) regional species pool and each local community.

For example, different forest dynamics can play an important role in determining forest diversity and hence the estimation of migration. Wood density, relative growth rate, and seed mass are related to dispersal, shade tolerance and are considered indicative for difference between pioneering or nonpioneering species (Hammond & Brown, 1995; Phillips, Hall, Gentry, Sawyer, & Vásquez, 1994; Seidler & Plotkin, 2006; ter Steege & Hammond, 2001; ter Steege et al., 2006). High wood density, slow growth, and large seed mass are reflected in slower forest dynamics (Malhi et al., 2004; Phillips et al., 2004). In contrast, low wood density, low seed mass, and faster turnover of individuals are reflecting faster forest dynamics. Marzluff and Dial (1991) showed that turnover and seed mass influence the ability to colonize new resources, leading to a potential higher diversity for forests having higher turnover and smaller seed mass. On the other hand, strong selective pressures or a very homogeneous environment in combination with fast turnover might cause plots to look more similar to each other due to natural selection, hence decreasing differences in species composition or even decreasing total species richness. In both cases, estimation of migration would potentially be relatively high as similarity between plots is also fairly high (low betadiversity), but again, neither neutral processes nor dispersal had little to do with it. Strong natural selection and a very heterogeneous habitat can also cause high betadiversity, decreasing estimates of migration. The above‐mentioned processes shape species composition and have an influence on the connection between the regional species pool and the local species pools, but have no neutral fundament. To be fair, the stochastic (neutral) counterpart of selection, ecological drift, can obviously also cause differences in species composition. Similar to population genetics, if drift is very pronounced, rare species will disappear and systems will lose diversity. But we know that this is by far not the only mechanism responsible for differences in community composition and that estimates of migration do not tell us specifically how much influence this stochastic mechanisms has in shaping diversity. Regarding this mechanism, we did observe an interesting pattern in the ratio between observed and expected singletons according to Fisher's logseries. As communities are structured according to Fisher's logseries, we can calculate the expected number of singletons based on the total number of species and individuals and compare this with the observed number of singletons in each sample. When forests are well mixed in the case of little dispersal limitation, the observed number of singletons should approach the expected number of singletons dependent on sample size. When this ratio deviates from one, this indicates that local plots are less connected to each other over larger distances resulting in a clumpier distribution. This eventually means fewer singletons than expected according to Fisher's logseries based on the number of individuals and species. We showed that there indeed was a strong relationship between the amount of migration from the metacommunity and this ratio of expected versus observed singletons. This idea is further explained and studied in the Supporting information (Appendix S4 Further analysis of migration using Fisher's logseries). A last note on interpreting estimates of migration focuses on the aspect of time. Given enough time on an ecological time scale, a collection of local communities will potentially have shared much of their species overall, even when having low direct migration between each local community. This is the result of each local community acting as a stepping stone, if individual species travel short distances each generation, they can still travel great distances. This inevitably increases the theoretical value of migration. Small differences in species composition (and thus high estimates of migration) can thus be the result either of low migration over a long period of time or high migration in a short period of time.

### 4.1 Reinterpreting estimation of migration from field data

We showed that estimates of migration from all three regions differed markedly (see Table 4). Although there were small differences between estimations when using different methods, relative differences between each dataset within one method were comparable. Guyana and Suriname showed the lowest migration probabilities, followed by French Guiana and finally Ecuador. Knowing that these estimates of migration are actually ecological aggregates, what differences in these forests can we attribute to these differences in migration probability? The relationship between community dynamics and alpha‐diversity was already shown for forests within Guyana (ter Steege & Hammond, 2001). Ter Steege et al. (2003) furthermore showed that on average, Western Amazonian forests are 150 individuals/ha denser than Eastern Amazonian forests and also have a higher alpha‐diversity. Forests of the Guiana Shield also experience a less heterogeneous environment and a more climax species composition having a higher seed mass and higher wood density in comparison with forests of Ecuador (ter Steege et al., 2006). This all suggests that forests of the Guiana Shield probably experience an overall stronger selection pressure, slower dynamics, and potentially also a higher impact of ecological drift due to smaller population sizes and less dispersal ability. All of these potentially lead to a stronger distance decay of similarity and a higher betadiversity, both also shown earlier (Pos et al., 2014). This would also explain a lower estimate of migration of forests of the Guiana Shield in comparison with Western Amazonian forests such as those of Ecuador.

## 5 Conclusions

We have shown that estimation of migration using methods based on species composition fails when estimating from spatially (semi‐)explicit simulations. Estimation was only correct when our spatially semi‐explicit model approached a spatially implicit world. We summarize that there are three major problems when using estimation methods based on spatially implicit models on a spatially explicit reality: (1) Estimations of migration relate to the differences in species diversity between plots and the diversity of the total sample used for analysis as it is based on a spatially implicit model, not an actual mechanism of dispersal; and (2) as differences in species diversity can be the result of a number of potential causes, the migration parameter does not solely reflect neutral dynamics as it is assumed to do so in neutral models. It is an aggregated ecological parameter, capturing a myriad of different processes. And (3) even if the migration parameter could actually be considered being reflective of the migration of individuals and not including any other mechanisms, these methods still only look at the “end result” of the homogenization. Hence, it does not shed any light on actual current forest dynamics, as it can be the effect of much migration in a short period or little migration over a long period.

The only method used in this study not based on species composition and hence not influenced by the problems mentioned above is the (corrected) plot geometry method by (Chisholm and Lichstein (2009). This uses plot geometry and absolute dispersal distances of individuals. It therefore attempts to estimate the actual amount of migration per time index as migration, although the original authors still implemented this into a spatially implicit model. For spatially (semi‐)explicit models, it offers a much more intuitive implementation of migration and shows promising results (ter Steege et al., in press). We propose that the next steps would be to study the real importance of migration implementing such a mechanistic estimate of dispersal into semispatially explicit models (Pos et al., in preparation). By doing so, we not only investigate the influence of dispersal directly but also have a more objective way to study the influence of neutral processes and to distinguish between sources of betadiversity. If dispersal would be the only mechanism driving diversity, such models should be able to predict community composition to a good degree. If not, then other mechanisms must be invoked. The interesting question is how this differs between different regions, for example, between more dynamic and slow forests such as Ecuador versus the Guyana Shield (Pos et al., in preparation). A different interesting question is regarding the influence of species richness and the ratio between species richness of the metacommunity and the local communities. Here, we focused on tropical forest systems as we have access to large‐scale datasets to test these models. But asking similar questions across multiple scales of diversity would most likely yield even more questions on the importance of regional diversity and the size of the species pool, which may prove a significant challenge.

Our main conclusion here is that spatially implicit models mimic the real world in a very good way simply because they make us of an aggregated ecological parameter, incorporating not only dispersal but everything determining the connection between a regional species pool and a local species pool. But the world simply is not spatially implicit; at least not for tropical tree species, and we should reject all inferences from such models on whether communities behave neutrally or not. Knowing this contains all possible filters that have been proposed, it does not further our knowledge of forest dynamics as we can only infer whether there is strong or weak filtering, it being either dispersal or establishment or both. Obviously, if we feed non‐neutral (assuming the real world is non‐neutral) data into a neutral model, models will still create output and methods for estimation of parameters will still generate parameter values. The importance, however, lies in the interpretation of these estimates. In neutral models, the emphasis lies on limited migration of individuals for explaining differences in composition. Many biologists thus interpret migration from such models as a mechanistic explanation for said differences. What we have tested here is whether this is reasonable or not and show that it is not and that we should be careful with these interpretations. As such, either assuming neutral dynamics or not, we cannot be sure what we are actually estimating from our spatially explicit world using methods based on species composition: low migration, high selective pressures, slow dynamics or fast dynamics, stronger drift, weak or strong natural selection, effects over short or long periods? The only thing we know is that we are estimating how much difference there is between the plots and the overall pool of diversity, and it is unlikely this is based solely on implicit neutral dynamics.

## Conflict of Interest

None declared.
