# Supplementary material for: Estimating and interpreting migration of Amazonian forests using spatially implicit and semi‐explicit neutral models
Source: Ecol Evol. 2017 May 5;7(12):4254–65. doi: 10.1002/ece3.2930 (PMC5478059; doi:10.1002/ece3.2930)
Supplement: Supplementary file 1 [file ECE3-7-4254-s001.docx]

SUPPLEMENTARY ONLINE MATERIAL

S1 Summary of used estimation methods

S2 Stabilization time and plot size of simulations

S3 A comment on the plot geometry multi subplot calculation

S4 Further analysis of migration using Fishers Log series S1 *Summary of used estimation methods*

We focus on five different parameter estimation methods 1) Etienne’s Sampling formula (Etienne 2005, 2009a), 2) the Inference method by Jabot et al (Jabot *et al.* 2008), 3) The G_st_ statistic adopted from population genetics (Munoz *et al.* 2008), 4) Etienne’s two-stage sampling (2009b) formula, which is an extension on the two-stage-estimation method by Munoz *et al.* (2007) and 5) a method by Chisholm & Lichstein (2009) based on plot geometry and absolute dispersal distances. Each method is summarized briefly below.

*Etienne’s sampling formulae*

Etienne (2005) presented a sampling formula for calculating the joint probability of species abundances in multiple local samples and its use to estimate migration. He based his work on the original spatially implicit version of the UNTB (Hubbell 2001, Volkov *et al.* 2003, Etienne 2005), where immigrants are drawn from a regional species pool according to one aggregated migration parameter *m* (Hubbell 2001; see also figure S1). This migration parameter was transformed into the dispersal number I_,_ which is related to sample size J and the migration probability *m* by $I=\frac{m(J-1)}{1-m}$ $I=\frac{m(J-1)}{1-m}$ showing that *m* is also related to sample size by$m=\frac{I}{(I+J-1)}$ $m=\frac{I}{(I+J-1)}$ (Etienne and Alonso 2005). Considering that each local community is a sample from the larger regional species pool we can imagine species entering the local community, establishing themselves and from that point forward potentially increasing in abundance by propagating (taking up the role as ancestors for all individuals of that same species in the future time). From the moment these ancestors arrive in the local community and going forward in time, each individual belonging to a specific species gives rise to a number of descendants being of the same species (as there is only speciation in the metacommunity), ultimately resulting in the species abundance distribution in the present community (Etienne 2005). If we would know all these intermediate steps (i.e. the number of individuals each ancestor of a specific species would have produced) it would be possible to derive what Etienne calls the “species-ancestry–abundance” distribution (D_+_) (Etienne 2005). As this is not possible in many cases, the current observed species abundance needs to be calculated as the sum of all possible species-ancestry-abundance distributions, which in turn is given by the multivariate probability P[D|θ*, m, J*] of observing a specific species abundance distribution (Etienne and Olff 2004). This multivariate probability is the likelihood *P* of observing a specific species abundance distribution *D* (for *S* species, *D= (n_1_,n_2_, …, n_S_)*) under the constraint of the suit of parameters used, thus P[D|θ*, m, J*], for the exact derivation of this expression, see Etienne & Olff 2004a,b. Although still rather complex, Etienne presented an simplification of this multivariate probability in 2005, which was incorporated in the freeware program Tetame <current version 2.1: http://chave.ups-tlse.fr/projects/tetame.htm>. Etienne’s estimation method of 2005, however, still has the assumption that all samples share a similar dispersal limitation. In 2007, Munoz *et al.* proposed a two-stage estimation method developed to circumvent problems encountered by the sampling formula of Etienne when dealing with small samples (Munoz *et al.* 2007). They first start by resampling a single individual from each separate sample (first stage) from which theta (*θ_meta_*) is then estimated using Ewens’ sampling formula (Ewens 1972). This procedure is repeated numerous times and the results averaged. The second stage of the estimation procedure is simply calculating the migration parameters *I* and *m* for each separate sample in the dataset using Etienne’s sampling formula as described above with the use of *θ_meta_* as *θ*. However, this approach has difficulties when estimating *θ* in the case of having either few samples or many samples that are very different from each other (Etienne 2009a). Later, Etienne therefore provided a renewed sampling formula as an improved two-stage estimation method, better suited for dealing with multiple samples having potentially very differing degrees of dispersal limitation (Etienne 2009b). This “two-stage Etienne estimation method” will also be used in the comparison; the coding for estimation was made available in the PARI/GP environment (“PARI/GP version 2.4.3” 2008) as appendix (Etienne 2009b). There were however a few practical issues while attempting to use the coding. Only older versions of PARI/GP could be used (pers.com Rampal Etienne) and calculation time was lengthy (taking approximately 4 days per subset to compute on a standard desktop computer with an Intel Core i5-4670 3.40 GHz processor and 8Gb RAM, which in total would mean roughly 200 days for all spatially implicit and the semi-explicit datasets). This in combination with the apparent small difference between the methods after performing the calculation on part of the data lead to the choice to use this method only for estimation of the field datasets and one spatially implicit dataset based on the empirical set of Ecuador.

*Inference method*

Jabot *et al.* (2008) used a different approach by using the pooled abundances of all species over all samples as regional species abundances to infer parameters for their model, rather than a summary based on the estimation of *θ_meta_*. They assumed that plots are randomly placed and not stratified to particular habitats. If this would be the case, then the estimate of migration would be biased, resulting from a non-random sampling of the regional species pool. Jabot *et al.* consider the regional relative abundances to be fixed, however, and by doing so ignore spatial aggregation of species. Using the constructed regional species pool, a maximization of the likelihood for the parameters to be estimated is achieved by optimization of their sampling formula (Jabot *et al.* 2008). This method is also available in the Tetame freeware program <current version 2.1: <http://chave.ups-tlse.fr/projects/tetame.htm>>.

*G_st_ statistic*

Munoz *et al.* (2008) proposed an estimation method based on the F_st_ statistic from population genetics. Originally, the G_st_ statistic as proposed by Nei in 1973 infers the extent of genetic differentiation between populations by comparing the variation in alleles among populations (i.e. betadiversity) and overall allelic diversity at the specific locus (gamma diversity). Nei’s genetic distance measure (Nei 1977, Takahata and Nei 1984) has assumptions comparable to Hubbell’s NT: all loci have the same rate of neutral mutation, there is a stable effective population size and this population is in a mutation versus drift equilibrium. Munoz *et al*. (2008) made a similar approach as Hubbell (2001) using theory from population genetics to estimate parameters for the neutral model. A very large set of alleles from a single locus is comparable to the number of species in large area. As such, the G_st_ statistic can be viewed as a measure of the variation among samples not in allelic diversity, but in species diversity. To estimate migration, three estimators of similarity are used: F_intra_ as the probability that two individuals in a sample (*k*) are conspecific, F_inter_ that two individuals one from sample k and one from sample l are conspecific and F_global_ that two individuals samples from the larger species pool are conspecific (Munoz *et al.* 2008). The average intrasample similarity ($\tilde{F}$_intra_) over all samples is related to Simpsons alpha diversity (Simpson 1949) as Div_α_ = 1-$\tilde{F}$_intra_, F_global_ is related to gamma diversity in a similar fashion as $\tilde{F}$_intra_ is to Div_α_ by Div_γ_ = 1-F_global_ (Munoz *et al.* 2008) and lastly, betadiversity as Div_β_ = $\tilde{F}$_intra_ - F_global_. These similarity statistics together form the Gst statistic for each separate sample k by $G_{st}\left( k \right)=\frac{F_{intra}\left( k \right)-F_{global}(k)}{1-F_{global}(k)}$. This relationship measures the relative dissimilarity between a sample and the regional species pool, which is considered to be the sum of all samples. Munoz *et al.* (2008) show that after derivation, the G_st_(k) is dependent only on the migration number I(k) when the sum of all individuals is much larger than that of a single sample. With this it is possible to estimate I(k) from each sample, under the assumption, however, that these samples have enough distance between them to actually be separate distinct local communities. For a detailed analysis of the exact and approximate estimators (sampling without and with replacement) and the relationship of the average betadiversity to similarity relative to a specific sample k, see Munoz *et al.* 2008.

*Chisholm & Lichstein’ 2009 plot geometry method*

All of the above described estimation methods lack a biological interpretation of the migration parameter. Chisholm and Lichstein (2009) presented expressions related to actual dispersal kernels and plot geometry for approximating the amount of migration, making it a more “biological intuitive” method. The main result of their efforts is their expression (2): $m \approx\frac{Pd}{\pi A}$, with *P* as the perimeter of the plot in meters, *d* the actual mean absolute dispersal distance in meters and *A* the total surface area of the plot in square meters. This expression holds only for large plots, as long as the size of the plot is relatively large in comparison to the mean dispersal distance it can be applied. They also show this for the case of a square plot and bivariate Gaussian kernel, yielding their expression (3): $m=1-\left\{ erf\left( \frac{w\surd\pi}{2d} \right)-\left( 1-exp\left[ -\frac{\pi w^{2}}{4d^{2}} \right] \right)\frac{2d}{\pi w} \right\}^{2}$. Where *erf* is an error function and *w* is the edge of the plot. As the size of the plot increases relative to the dispersal distance *d* the *erf* and *exp* function tend to 1 and 0 respectively, resulting in the same expression as (2). As example, for a square plot with an edge length of 100 meters and mean dispersal distance of 30 meters, the error function becomes .999 and the exponent becomes 1.6e^-4^, thus becoming negligible and resulting in the general solution of expression (2) (Chisholm & Lichstein 2009). In order for the *erf* function to be >.99 and the *exp* function to be <.025 the ratio $\frac{d}{w}$ $\frac{d}{w}$ $\frac{d}{w}$ should be at least < .45 meaning that the edge of the plot size should roughly be at least twice the mean dispersal distance. For this study, when this was the case expression (2) was used to approximate *m*, otherwise expression (3) was used for approximating the migration parameter. This method does suffer from a serious drawback: it only takes into account the number of immigrants arriving in the local community from outside of a plot as potential replacements. Recruits, however, can come from inside the local community as well. When for instance the replacement falls on the edge of a plot, assuming a Gaussian dispersal kernel, the probability distribution is symmetric. To solve this, we used a corrected version of the Plot Geometry method; see the Supplementary Online Material (S3).

S2. *Stabilization time and plot size of simulations*

Two major components determine whether simulations stabilize in terms of their species abundance distribution: size of the plot and runtime of the simulation. To determine both we made use of Fisher’s Logseries distribution (Fisher et al. 1943). To test whether time and plot size was adequate a simulation was run with and without dispersal limitation. As the local communities start out as a random sample of the metacommunity, in the latter case they reflect the composition of the metacommunity. The number of singletons and Fisher’s alpha is then expected to be almost identical to that of the metacommunity given a large enough sample due to random variation of the sampling procedure. According to Fisher’s logseries the total number of individuals (N_T_) found in any community can be calculated by N_T_ = S_1_ + 2S_2_ + 3S_3_ + 4S_4_ + iS_i_ with S_i_ being the abundance class a species belongs to (i.e. S_1_ are all singletons, S_2_ all doubletons etc.). In terms of Fisher’s parameters *α* and *x* this is equal to N_t_ = αx + αx^2^ + αx^3^ + αx^4^ + αx^i^, where αx is the number of species predicted to have only 1 individual (singletons), αx^2^ /2 the number of doubleton species etc. Assuming x is a value between 0 and 1, Fisher’s logseries approaches a geometric series given by 1/(1-x). N_t_ then becomes N_t_ = αx(1/(1-x)) = αx/(1-x). From this we can derive the value of x using N_T_ (1-x) = αx, which leads to N_T_ = x(α+N_T_) and finally to x = N_T_/( N_T_+α). By plugging this into the first term of the logseries we come up with the equation for the expected number of singletons in the community, Φ_1exp_ = αx = αN_T_/(N_T_+α). By calculating the expected number of singletons (Φ_1exp_) for any given community given the total community size and comparing this with the actual number of singletons observed of the sample (Φ_1obs_) in the case without dispersal limitation we can test whether sample size was adequate. To test generation time for scenario’s with dispersal limitation we simulated a set of 25 local communities, each having 625 individuals starting out as a random sample of the metacommunity. Migration was set to an arbitrary value of 0.5, meaning half of replacements is coming out of the metacommunity and half are local recruits (as this is a spatially implicit model). After the initial sampling, at some point local communities running neutral dynamics should stabilize in terms of the species abundance distribution and the difference between Fisher’s alpha of the previous generation and the next generation should become negligible (Figure S3).

*Simulated metacommunities and stabilization of the sampling procedure*

Construction of the MC-Low yielded 20,191,600,511 individuals distributed over 4,582 species with a Fisher’s alpha of 251. For the MC-High this yielded 5,611,001,426 individuals belonging to 6,834 species, with a Fisher’s alpha of 416 (Figure S2). Analysis of the stabilization for sampling each of the simulated spatially implicit datasets showed that approximately 625 individuals and 1e^5^ sampling rounds was sufficient to minimize the difference between Φ_1exp_ and Φ_1obs_ (Figure S3).

*S3 A comment on the plot geometry multi subplot calculation*

According to Chisholm & Lichstein (2009), their Plot Geometry method also allows estimation of migration for disconnected local communities. As the direct dispersal between distant subplots becomes negligible, approximately at twice the mean dispersal distance according to their simulations they state the migration parameter can be calculated by $m= \sum_{i=1}^{K} P_{i}\hat{m}_{i}$, with *K* the number of subplot, *p_i_* as the probability of death occuring in subplot *i* and $\hat{m}$_i_ the probability that the parent of the replacement has an origin outside this or any subplot in the total community. Because direct dispersal between subplots at a certain distance becomes negligible, migration for each subplot can be approximated by assuming each plot is completely isolated and therefore estimation can be performed by using expression 3 of Chisholm & Lichstein (2009) which states $m=1-\left\{ erf\left( \frac{w\surd\pi}{2d} \right)-\left( 1-exp\left[ -\frac{\pi w^{2}}{4d^{2}} \right] \right)\frac{2d}{\pi w} \right\}^{2}$. In addition, because under the assumptions of the UNTB (Hubbell 2001) the probability of a death of a particular individual is simply proportional to the relative abundance of the species it belongs to in that subplot, the approximation for migration becomes $m= \frac{1}{J}\sum_{i=1}^{K} J_{i}m_{i}$. They continue by stating that when there are many disconnected subplots, the assumption of panmixis of the original UNTB might be a potential violation but that despite this shortcoming, their method still remains useful as instead of simulating a single migration parameter you could calculate it for each separate subplot. However, we fail to see the added value of this last statement, as $\sum J_{i}=J$ and $\frac{\sum J_{i}}{J}=1$, *m* reduces to $m=m_{i}$, meaning that assuming the plot geometry and mean dispersal distance for each plot is constant, one could simply calculate it for one plot and it would be the same for all plots. Only in the case where each subplot has different geometry or mean dispersal distance this has merit.

*A correction for the plot geometry method*

Chisholm & Lichstein (2009) proposed an estimation method based on simple plot geometry and the mean dispersal distance of individuals. They assume an infinite two-dimensional landscape on which a quadrat of area *A* is “thrown down”, being the local community (LC). The LC then consists of *J* individuals, calculated as the density of individuals in the infinite landscape (*ρ*) captured within the area *A*. Similar to Hubbell’s UNTB (Hubbell, 2001), individuals within the LC die and are being replaced at random from either parents within the LC (local recruitment) or from outside of the LC (migrants). Each individual, either within or outside the local community is thus capable of producing offspring. In contrast with the original UNTB, Chisholm & Lichstein incorporate space by assuming the offspring of each parent has the ability to disperse according to a radially symmetric dispersal kernel, assuming there is no difference between individuals. Imagine the LC is divided into x_n_ by y_n_ gridcells of 1 m^2^ with *n* the edge length of the area *A*. Each cell is occupied by an individual and each timestep an individual dies, vacating one of the grid cells with coordinate (x_i_, y_i_). Chisholm & Lichstein consider the location of the parent individual for the replacement randomly selected from a dispersal kernel, which is centered at the dying individual. It then is the mean dispersal distance of individuals, in combination with the location (x_i_, y_i_) that determine the overall probability of a replacement coming from either inside or outside the local community.

For replacements near the edge of the plot area *A*, this latter probability is the largest considering half of the kernel is situated outside of the plot, rather then inside (figure S4_A_). They justify using the replacement location as the center considering the kernels are symmetrical from either perspective and the result will be the same when you consider it from the parent perspective.

Because they calculate it from the perspective of selecting the parent from the dispersal kernel centered at the replacement location, they only take into account a small fraction of potential recruits from both within and outside of the plot. In this case indeed, swopping from either the parent or the replacement perspective does not matter because they are symmetrical (figure S4_B,C_). The consequence however also is that the probability kernel only is calculated with a maximum mean μ as the edge of the plot (L in the figures S4_A_-_D_), because a replacement location cannot be outside of the surface area *A*. If we however look from the perspective of the potential parents, considering a dispersal kernel with a mean dispersal distance (σ) of 20 meters the entire probability density curve has a range of approximately +-σ4 from the edge of the plot (figure S4_D_, in this case with a mean dispersal distance of 20 meters this would be the edge +- 80 meters). This in turn means that parents within the range of L+- 4σ can potentially supply the replacement (figure S4_D_). We therefore simulated all dispersal kernels for each meter in the range L+-4σ and calculated the entire surface area’s of the probability density kernels coming from both inside the plot (figure S4_D_, red) and outside (blue) and divided the surface area of the blue density kernels by the total, yielding the ratio of replacements being a migrant from outside the plot. Disregarding the mean dispersal distance σ, this is always approximately 30%, meaning that from assuming an individual every meter, the ratio of migrants of the total estimated migration by the Plot Geometry method should be multiplied with a correction of .3. As is shown in the main text, using this corrected migration parameter to simulate a spatially implicit forest shows this has extremely good fits with the actual field data.

S4 *Further analysis of migration using Fishers Log series*

Although estimation methods were only able to estimate joint migration rate and only accurately for the limitation of *m.meta* > *m.adj*, we did observe an important pattern in the amount of singletons per plot and in the total simulated forest. The amount of singletons was strongly dependent on the amount of migration from either source, adjacent or the metacommunity. An increase in the probability of migration from the metacommunity was being reflected in a relative high number of singletons. In contrast, this was relatively low when migration was mostly coming from adjacent plots. Interestingly, this pattern is comparable to the amount of rare alleles found in population genetics in relation to genetic drift, which is also dependent on the amount of migration. Disregarding selective pressures, when populations experience little to none migration of individuals, they will eventually become fixated for specific alleles due to sampling error. Following a similar train of thought, due to the probabilistic nature of sampling in neutral theory models, singletons are lost and are not being replaced from the metacommunity but by the more common species from adjacent plots. In this case, the more common species increase in abundance as they are shared more and more among adjacent plots, hence the amount of singletons will decrease. We can use this pattern to shed light on whether much migration is coming from adjacent localities or from the main source pool of species. As communities are structured according to Fisher’s Logseries (Fisher et al. 1943), we can calculate the expected number of singletons (Φ_1exp_) based on the total number of species and individuals and compare this with the observed number of singletons in the samples (Φ_1obs_) if forests follow true neutral dynamics. A low singleton ratio of Φ_1obs_/ Φ_1exp_ is expected when most migrants are from adjacent plots (leading to clumped patterns of species composition). When the ratio approaches 1 this indicates that most migrants are originating from the metacommunity, ultimately representing a homogeneous random sample of the metacommunity when all migrants actually do originate from the metacommunity. We tested this for all combinations of migration used in the spatially semi-explicit simulations and indeed found that a higher ratio was indicative of a higher migration probability from the metacommunity (Figure S7). We also applied this to the field data. For each dataset, Φ_1exp_ was calculated for each plot and compared with Φ_1obs_. Results showed that for all datasets there was on average a higher than 1 ratio of observed versus expected singletons (Figure S8 and Table S2). This means that on average, plots have more singletons than would be expected purely on the logseries and neutral dynamics. As is explained in the main text, this might be explained by the fact that these forests indeed experience more than just neutral dynamics. Perhaps there is strong selection forcing species on their way out more than we would expect on the basis of stochastic processes alone or differential selection might allow specialists to persevere in low numbers.

REFERENCES:

Chisholm, R. A., and J. W. Lichstein. 2009. Linking dispersal, immigration and scale in the neutral theory of biodiversity. Ecology Letters 12:1385–1393.

Etienne, R. 2005. A new sampling formula for neutral biodiversity. Ecology letters:253–260.

Etienne, R. 2009. Improved estimation of neutral model parameters for multiple samples with different degrees of dispersal limitation. Ecology 90:847–852.

Etienne, R., and D. Alonso. 2005. A dispersal-limited sampling theory for species and alleles. Ecology Letters:1147–1156.

Etienne, R., and H. Olff. 2004. A novel genealogical approach to neutral biodiversity theory. Ecology Letters:170–175.

Fisher, R., A. Corbet, and C. Williams. 1943. The relation between number of species and the number of individuals in a random sample of an animal population. The Journal of Animal Ecology.

Hubbell, S. P. 2001. The uniﬁed neutral theory of biodiversity and biogeography. Princeton Monographs in Population Biology. Princeton University Press, Princeton, New Jersey, USA.

Jabot, F., R. Etienne, and J. Chave. 2008. Reconciling neutral community models and environmental filtering: theory and an empirical test. Oikos.

Munoz, F., P. Couteron, B. Ramesh, and R. Etienne. 2007. ESTIMATING PARAMETERS OF NEUTRAL COMMUNITIES: FROM ONE SINGLE LARGE TO SEVERAL SMALL SAMPLES. Ecology 88:2482–2488.

Munoz, F., P. Couteron, and B. R. Ramesh. 2008. Beta diversity in spatially implicit neutral models: a new way to assess species migration. The American naturalist 172:116–27.

Nei, M. 1977. F-statistics and analysis of gene diversity in subdivided populations. Annals of human genetics 41:225–233.

PARI/GP version 2.4.3. 2008. . manual, Bordeaux.

Simpson, E. 1949. Measurement of diversity. Nature 163:1949.

Takahata, N., and M. Nei. 1984. FST and GST statistics in the finite island model. Genetics 11:349–352.

Volkov, I., J. R. Banavar, S. P. Hubbell, and A. Maritan. 2003. Neutral theory and relative species abundance in ecology. Nature 424:1035–7.

Table S1. Estimates of migration from spatially implicit simulated datasets based on the MC-low and MC-high metacommunities. For each dataset created, the runtime and number of plots are indicated. Size was set on 625 individuals per plot and runtime was 1e5 generations, based on stabilization time of the sampling procedure as explained in the main text. Species richness depends on both runtime and migration. Estimated migration parameters are shown with standard deviation of the mean calculated for all plots.

| GS based (MC-Low, 67 plots) | | | | | | | | | | |
| --- | --- | --- | --- | --- | --- | --- | --- | --- | --- | --- |
| Simulation | | | | | Estimated Migration | | | | | |
|  | | | | | One-stage est. | | Inference method | | G_st_-statistic | |
| dataset | Nr. sp. | Nr. sing | m |  | m1 | SD | m2 | SD | m3 | SD |
| 1 | 1062 | 131 | .21 |  | .231 | .032 | .219 | .015 | .211 | .043 |
| 2 | 1100 | 151 | .25 |  | .250 | .032 | .255 | .015 | .247 | .059 |
| 3 | 1130 | 163 | .31 |  | .302 | .036 | .314 | .015 | .297 | .077 |
| 4 | 1155 | 157 | .35 |  | .294 | .034 | .359 | .016 | .341 | .095 |
| 5 | 1166 | 164 | .41 |  | .433 | .048 | .415 | .015 | .394 | .120 |
| 6 | 1199 | 172 | .45 |  | .443 | .051 | .458 | .015 | .430 | .141 |
| 7 | 1213 | 185 | .51 |  | .522 | .060 | .515 | .015 | .487 | .170 |
| 8 | 1220 | 196 | .55 |  | .553 | .054 | .553 | .014 | .521 | .198 |
| 9 | 1246 | 211 | .61 |  | .543 | .047 | .628 | .013 | .574 | .216 |
| 10 | 1253 | 201 | .71 |  | .586 | .046 | .717 | .011 | .670 | .289 |
| 11 | 1259 | 211 | .81 |  | .651 | .047 | .820 | .009 | .780 | .382 |

| FG based (MC-Low, 63 plots) | | | | | | | | | | |
| --- | --- | --- | --- | --- | --- | --- | --- | --- | --- | --- |
| Simulation | | | | | Estimated Migration | | | | | |
|  | | | | | One-stage est. | | Inference method | | G_st_-statistic | |
| dataset | Nr. sp. | Nr. sing | m |  | m1 | SD | m2 | SD | m3 | SD |
| 1 | 1077 | 141 | .21 |  | .238 | .033 | .216 | .015 | .209 | .044 |
| 2 | 1081 | 145 | .25 |  | .287 | .034 | .254 | .015 | .247 | .0459 |
| 3 | 1109 | 140 | .31 |  | .313 | .039 | .320 | .016 | .311 | .0770 |
| 4 | 1143 | 166 | .35 |  | .339 | .042 | .351 | .016 | .327 | .075 |
| 5 | 1164 | 158 | .41 |  | .427 | .052 | .413 | .015 | .386 | .099 |
| 6 | 1152 | 177 | .45 |  | .440 | .049 | .461 | .015 | .429 | .125 |
| 7 | 1169 | 182 | .51 |  | .478 | .058 | .530 | .015 | .489 | .153 |
| 8 | 1209 | 162 | .55 |  | .557 | .058 | .551 | .014 | .509 | .169 |
| 9 | 1212 | 193 | .61 |  | .578 | .060 | .624 | .013 | .588 | .220 |
| 10 | 1241 | 213 | .71 |  | .542 | .043 | .712 | .012 | .653 | .257 |
| 11 | 1254 | 224 | .81 |  | .375 | .059 | .824 | .009 | .760 | .325 |

| EC based (MC-High, 72 plots) | | | | | | | | | | | | |
| --- | --- | --- | --- | --- | --- | --- | --- | --- | --- | --- | --- | --- |
| Simulation | | | | | Estimated Migration | | | | | | | |
|  | | | | | One-stage est. | | Inference method | | G_st_-statistic | | 2-stage (Etienne) | |
| dataset | Nr. sp. | Nr. sing | m |  | m1 | SD | m2 | SD | m3 | SD | m4 | SD |
| 1 | 1589 | 205 | .21 |  | .280 | .025 | .2154 | .014 | .205 | .043 | .204 | .017 |
| 2 | 1659 | 244 | .25 |  | .264 | .030 | .254 | .014 | .241 | .056 | .245 | .022 |
| 3 | 1699 | 249 | .31 |  | .469 | .053 | .313 | .014 | .296 | .075 | .308 | .022 |
| 4 | 1718 | 266 | .35 |  | .528 | .055 | .359 | .014 | .336 | .096 | .344 | .025 |
| 5 | 1736 | 256 | .41 |  | .620 | .060 | .419 | .013 | .399 | .132 | .417 | .030 |
| 6 | 1776 | 297 | .45 |  | .648 | .061 | .460 | .013 | .435 | .157 | .449 | .036 |
| 7 | 1803 | 308 | .51 |  | .665 | .060 | .510 | .013 | .472 | .173 | .496 | .029 |
| 8 | 1804 | 283 | .55 |  | .712 | .053 | .556 | .013 | .525 | .212 | .547 | .040 |
| 9 | 1852 | 336 | .61 |  | .712 | .046 | .618 | .018 | .587 | .250 | .610 | .041 |
| 10 | 1891 | 368 | .71 |  | .729 | .036 | .724 | .010 | .695 | .326 | .713 | .046 |
| 11 | 1922 | 371 | .81 |  | .625 | .022 | .819 | .008 | .808 | .418 | .810 | .045 |

Table S2. Results belonging to figure S7 with mean Φ_1obs_ and Φ_1exp_ for all plots of the datasets Guyana/Suriname (GS; 67), French Guiana (FG; 63) and Ecuador (EC; 72).

|  | *Mean FA* | *Mean Nr. species* | *Mean ϕ*_1_*obs* | *Mean ϕ*_1_*exp* | *Ratio* | *Sd. ratio* |
| --- | --- | --- | --- | --- | --- | --- |
| GS | 20 | 58 | 22 | 19 | 1.198 | .33 |
| FG | 67 | 142 | 69 | 59 | 1.169 | .11 |
| EC | 78 | 146 | 75 | 68 | 1.115 | .17 |

**Figure S1.** Schematic view of the two spatio-temporal communities and their connection as implemented in the original Unified Neutral Theory of Biodiversity and Biogeography by Stephen Hubbell (2001)

**Figure S2**. Rank abundance curve for both MC-low (left) and MC-high (right). Numbers are based estimates for the number of individuals and species from (ter Steege et al 2013).

**Figure S3**. Stabilization time of the sampling model without dispersal limitation (m=1) based on both sample size (x^1^ axis above, red label) and number of generations (x^2^ axis below, black label). On the y-axis is the difference between the expected number of singletons (F1) and observed F1, which was calculated using Fisher’s Logseries.

**Figure S4**. Different situations of the distribution of Probability Density Kernels following a Guassian Distribution with *L* the edge of a plot and *d* the mean dispersal distance used as the standard deviation (σ) of the distribution and range given by *L* +- *4σ* to ensure a full range (as 3*σ* covers 99.73% of the values around the mean). A) A single dispersal kernel for propagules produced by a parent on the edge of the plot, B) All dispersal kernels from parents residing within the plot, C) All dispersal kernels from parents residing outside the plot and D) The combined plot of B and C.

**Figure S5**. Rank abundance curves (Guyana/Suriname top, French Guiana middle, Ecuador bottom) for the simulated datasets (red) in comparison with those of the actual field data plotted in the same graph (blue). Migration parameter used for sampling the simulated sets is based on the Gst statistic (left) and Inference method (right).

**Figure S6**. Rank abundance curves (Guyana/Suriname top, French Guiana middle, Ecuador bottom) for the simulated datasets (red) in comparison with those of the actual field data plotted in the same graph (blue). Migration parameter used for sampling the simulated sets is based on Etienne’s two-stage estimation method (left) and the corrected Plot Geometry method by Chisholm & Lichstein (right).

**Figure S7.** The calculated ratio of Φ_1obs_/Φ_1exp_ plotted against the given migration probability from the metacommunity (*m.meta*) in the semi spatially explicit model as described in the Supplementary Online Material. Dashed lines indicate the 95% confidence interval for the loess model.

**Figure S8.** Calculation of the mean ratio of observed singletons versus the expected amount of singletons per field dataset as explained in the main text of the Supplementary Online Material. Error bars shown are the standard deviations of the mean.

**Figure S9.** Schematic view of how both environmental distance and geographical distance can cause similar patterns in differentiation of species composition. Although these two mechanisms are each others opposite in terms of the niche versus neutral discussion, spatially implicit neutral models cannot differentiate between the two mechanisms responsible.

FIGURE S1


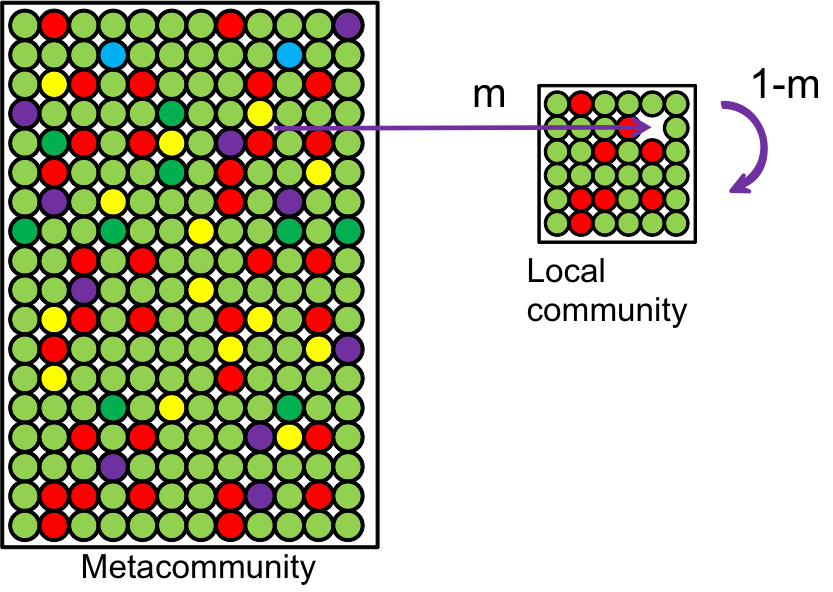
FIGURE S2

FIGURE S3


FIGURE S4

A

B
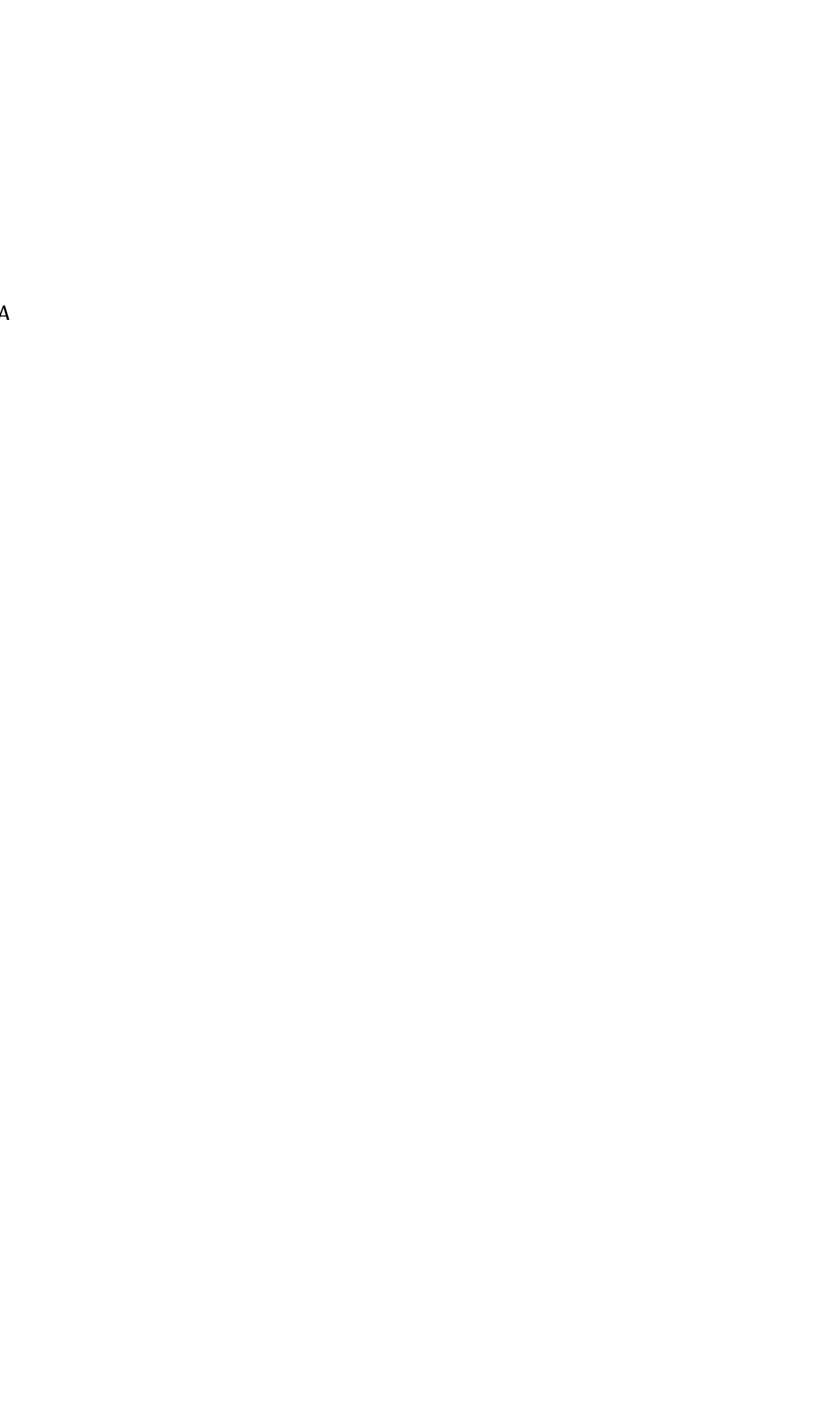


C
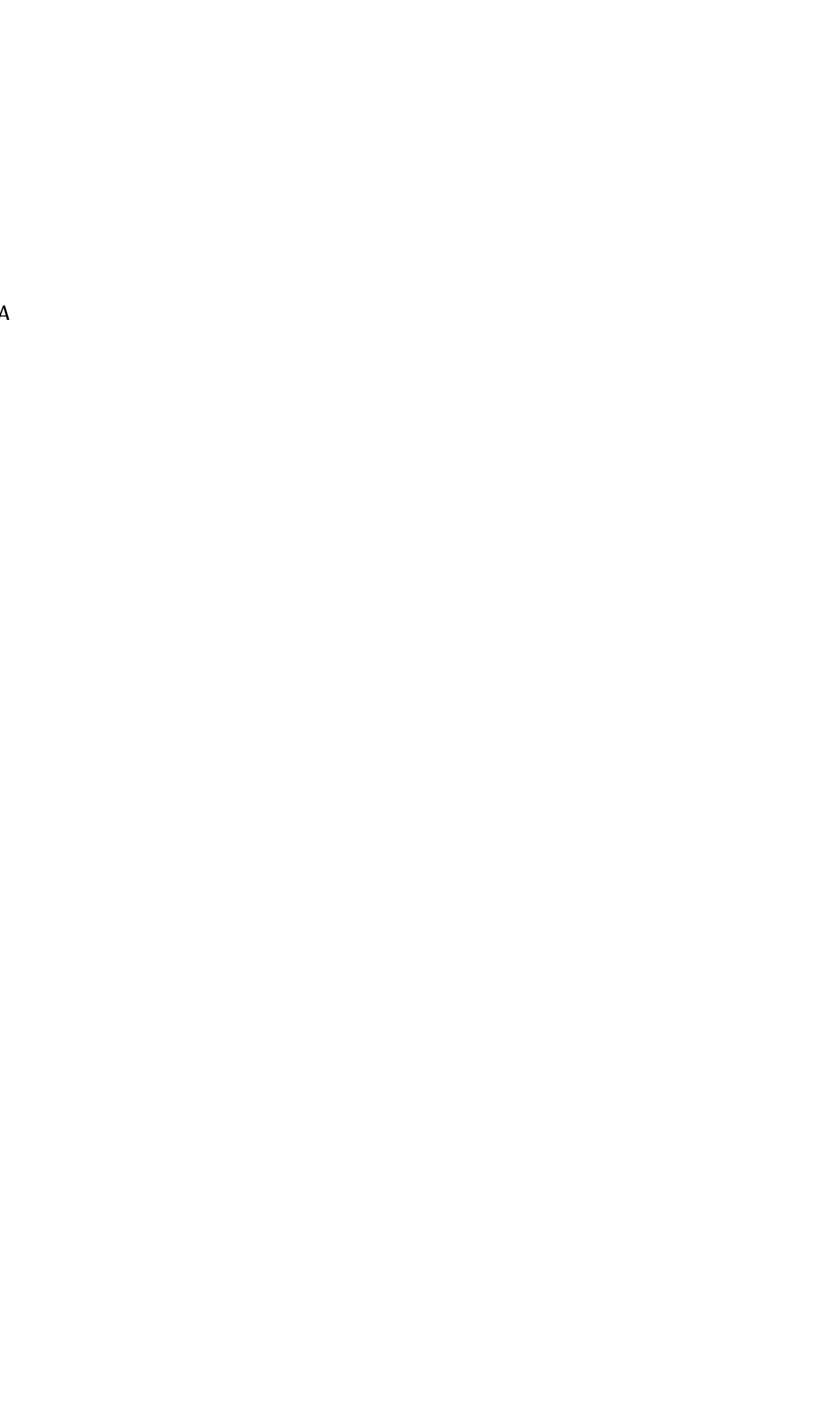


D
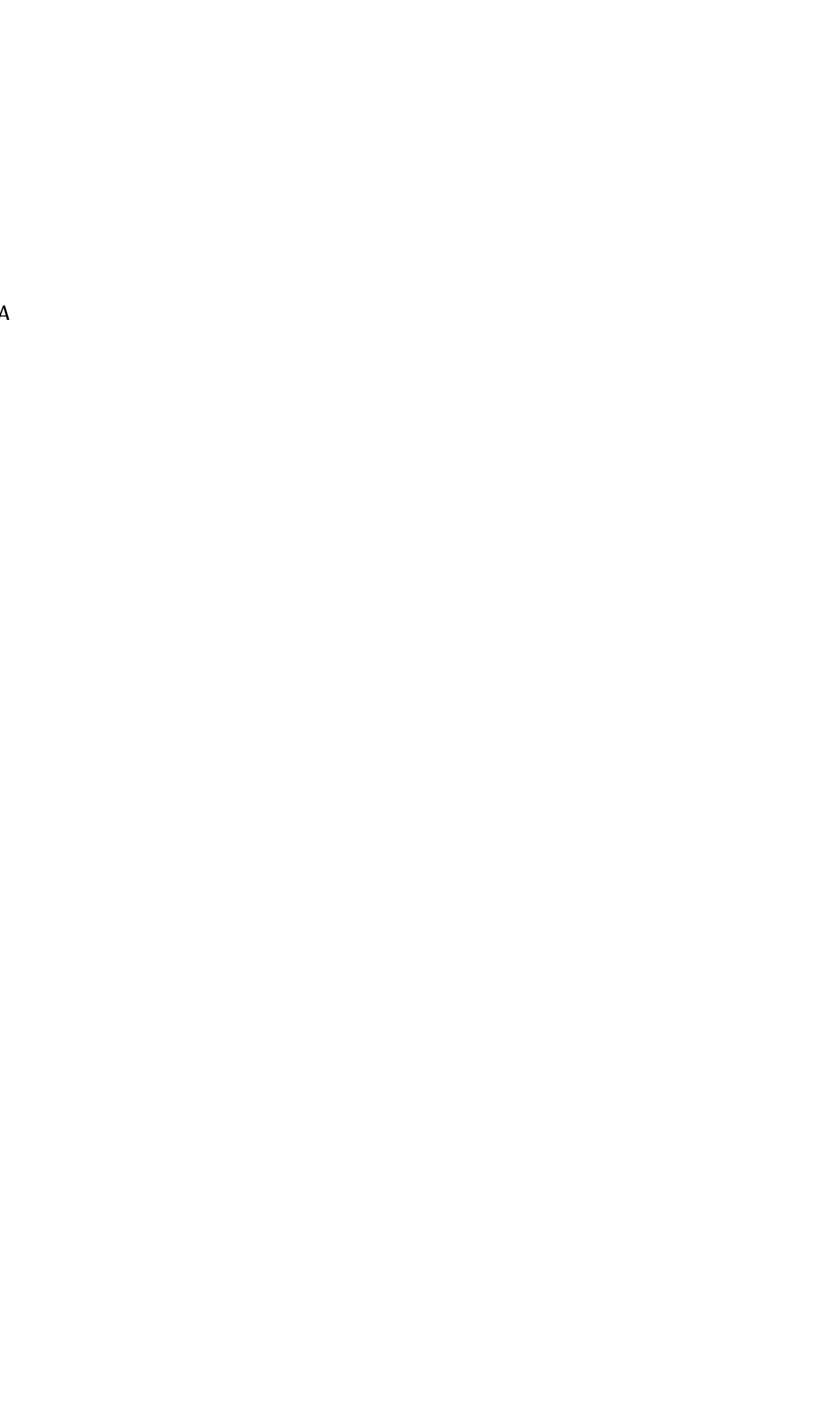


FIGURE S5

FIGURE S6

FIGURE S7

FIGURE S8

FIGURE S9

Environmental distance

Geographical distance

High similarity

Low betadiversity

**High m estimates**

Low similarity

High betadiversity

**Low m estimates**

Two different explanations for the same observed pattern. Either environmental distance or geographical distance can cause the same differentiation in species composition. Estimates of m however will be the same for both scenarios
